# On the reliability and validity of manual muscle testing: a literature review

**DOI:** 10.1186/1746-1340-15-4

**Published:** 2007-03-06

**Authors:** Scott C Cuthbert, George J Goodheart

**Affiliations:** 1 Chiropractic Health Center, 255 West Abriendo Avenue, Pueblo, CO 81004, USA; 2 Goodheart Zatkin Hack and Associates, 20567 Mack Avenue, Grosse Pointe Woods, MI 48236-1655, USA

## Abstract

### Introduction

A body of basic science and clinical research has been generated on the manual muscle test (MMT) since its first peer-reviewed publication in 1915. The aim of this report is to provide an historical overview, literature review, description, synthesis and critique of the reliability and validity of MMT in the evaluation of the musculoskeletal and nervous systems.

### Methods

Online resources were searched including Pubmed and CINAHL (each from inception to June 2006). The search terms *manual muscle testing *or *manual muscle test *were used. Relevant peer-reviewed studies, commentaries, and reviews were selected. The two reviewers assessed data quality independently, with selection standards based on predefined methodologic criteria. Studies of MMT were categorized by research content type: inter- and intra-examiner reliability studies, and construct, content, concurrent and predictive validity studies. Each study was reviewed in terms of its quality and contribution to knowledge regarding MMT, and its findings presented.

### Results

More than 100 studies related to MMT and the applied kinesiology chiropractic technique (AK) that employs MMT in its methodology were reviewed, including studies on the clinical efficacy of MMT in the diagnosis of patients with symptomatology. With regard to analysis there is evidence for good reliability and validity in the use of MMT for patients with neuromusculoskeletal dysfunction. The observational cohort studies demonstrated good external and internal validity, and the 12 randomized controlled trials (RCTs) that were reviewed show that MMT findings were not dependent upon examiner bias.

### Conclusion

The MMT employed by chiropractors, physical therapists, and neurologists was shown to be a clinically useful tool, but its ultimate scientific validation and application requires testing that employs sophisticated research models in the areas of neurophysiology, biomechanics, RCTs, and statistical analysis.

## Review

The role of the muscle system in spinal function has become increasingly well acknowledged. Manual muscle testing (MMT) as a method of diagnosis for spinal dysfunction has not been well utilized. This paper will present evidence that the MMT can be a legitimate and useful evaluation tool for the assessment of the musculoskeletal and nervous systems.

There are many ways of examining the nervous system and the musculoskeletal system. It has been proposed that the term neuromusculoskeletal system be adopted because examination of the one may reflect the status of the other [1,2]. The evaluation methods of many manipulative therapists often focus at either end of the nervous system, and this paper suggests that MMT provides a method of examining both (the central and the peripheral) ends.

MMT is the most commonly used method for documenting impairments in muscle strength. Limited muscle testing methods are taught in a number of chiropractic schools around the world, however in 2006 a major "stand alone" chiropractic technique that employs MMT for the evaluation of patients known as applied kinesiology chiropractic technique (AK), turned 42 years old. We propose in this review to look at the research status of MMT in the manual examination of the nervous system's status. The early years of the AK method are related elsewhere in detail [3]. The specific protocols and clinical objectives of the technique have been described in previous publications [3-9].

AK has therefore been used by a proportion of the chiropractic profession for over 42 years and is now used by other healing professions. In a survey by the National Board of Chiropractic Examiners in 2000, 43.2% of respondents stated that they used applied kinesiology in their practices, up from 37.2% of respondents who reported they used AK in 1991, [10-12] with similar numbers reported in Australia [13]. The general public's awareness of MMT and AK has also been increased worldwide by virtue of the patient education program Touch for Health (T4H) designed by an International College of Applied Kinesiology (ICAK) diplomate, John Thie. T4H was one of the first public self-help programs and there are claims that it is the fastest growing "body work" program in the world, used by over 10 million people [14].

For the purposes of this review we define MMT as a diagnostic tool and AK as a system for its use and therapy based on the findings of the MMT

In this paper we pose the following questions: 1) "Is the MMT approach worthy of scientific merit?" and 2) "How can new diagnostic and treatment techniques employing MMT be critiqued for scientific merit?" and 3) "Does this evidence add scientific support to chiropractic techniques (such as AK) that employ the MMT?"

Another main objective of this literature review was to investigate the evidence for intraexaminer reliability, interexaminer reliability, and validity of MMT in the assessment of patients.

## Methods

Online resources were searched using Pubmed and CINAHL (Cumulative Index to Nursing and Allied Health literature). The search terms "manual muscle test", "manual muscle testing", and "applied kinesiology" found over 100 articles in which the MMT was used to document strength in patients with 17 (primarily pain related) diseases/disorders, ranging from low back pain and sacroiliac joint pain to neck pain, post-whiplash syndrome, knee, foot, and shoulder pain, and included MMT for the evaluation of patients with post-polio syndrome, amyotrophic lateral sclerosis, muscular dystrophy, cerebral palsy, Down syndrome, mastalgia, hypothyroidism, dysinsulinism, enuresis and several other disorders of childhood.

After abstracts were selected for relevance and the papers acquired and reviewed, the literature was sorted according to relevance and quality. Inclusion criteria were that the report had a Cohen's kappa coefficient of 0.50 or higher (the magnitude of the effect size shown in the study to be significant) in regards to the intra- and inter-examiner reliability, and/or the validity (construct and content validity, convergent and discriminant validity, concurrent and predictive validity). This selection criteria is consistent with the one suggested by Swinkels et al for the evaluation of the quality of research literature [15]. Randomized clinical trials (n = 12), prospective cohort studies (n = 26), retrospective studies (n = 17), cross-sectional studies (n = 26), case control studies (n = 10), and single-subject case series and case reports (n = 19) were the types of studies reviewed. Studies with a control group (a randomized clinical trial), examiner blinding, and pre- and post-test design are indicated in the descriptions of each study. Duplicates and articles published in non-peer-reviewed literature were excluded.

Statistical presentations of the data are presented showing the average correlation coefficients of MMT examination upon the different patient populations for each study.

### Operational Definitions and History of the Manual Muscle Test

In order to be meaningful, all measurements must be based on some type of operational definition. An operational definition is a description of the methods, tools, and procedures required to make an observation (i.e. a definition that is specific and allows objective measurement). Kaminsky and Fletcher et al provide clinicians with some strategies to critically analyze the scientific merit of manual therapies [16,17].

A basic understanding of operational definitions is required in order to make judgments about the methods used in articles and to know which research findings should be implemented in practice. For example, how should we judge the value of the MMT for the gluteus maximus or gluteus medius muscles in cases of sacroiliac joint pain and dysfunction, knowing that statements range from "weakness of the gluteals is usually present in dysfunction of the sacroiliac joint" (Janda 1964) [18] to "the results of this study cast doubt on the suitability of manual muscle testing as a screening test for strength impairments"? (Bohannon 2005) [19].

Within the chiropractic profession, the ICAK has established an operational definition for the use of the MMT:

"Manual muscle tests evaluate the ability of the nervous system to adapt the muscle to meet the changing pressure of the examiner's test. This requires that the examiner be trained in the anatomy, physiology, and neurology of muscle function. The action of the muscle being tested, as well as the role of synergistic muscles, must be understood. Manual muscle testing is both a science and an art. To achieve accurate results, muscle tests must be performed according to a precise testing protocol. The following factors must be carefully considered when testing muscles in clinical and research settings:

• Proper positioning so the test muscle is the prime mover

• Adequate stabilization of regional anatomy

• Observation of the manner in which the patient or subject assumes and maintains the test position

• Observation of the manner in which the patient or subject performs the test

• Consistent timing, pressure, and position

• Avoidance of preconceived impressions regarding the test outcome

• Nonpainful contacts – nonpainful execution of the test

• Contraindications due to age, debilitative disease, acute pain, and local pathology or inflammation"

In physical therapy research, the "break test" is the procedure most commonly used for MMT, and it has been extensively studied [20-22]. This method of MMT is also the main test used in chiropractic, developed originally from the work of Kendall and Kendall [21,23].

In physical therapy the "break test" has the following operational definition [20-22]. The subject is instructed to contract the tested muscle maximally in the vector that "isolates" the muscle. The examiner resists this pressure until the examiner detects no increase in force against his hand. At this point an additional small force is exerted at a tangent to the arc created by the body part being tested. The initial increase of force up to a maximum voluntary strength does not exceed 1 sec., and the increase of pressure applied by the examiner does not exceed a 1-second duration. "Strong" muscles are defined as those that are able to adapt to the additional force and maintain their contraction with no weakening effect. "Weak" muscles are defined as those unable to adapt to the slight increase in pressure, i.e., the muscle suddenly becomes unable to resist the test pressure.

For example in the seated test for the rectus femoris muscle, a seated subject is asked to flex his knee toward his chest 10 degrees; when that position is reached, the examiner applies resistance at the knee, trying to force the hip to "break" its hold and move the knee downward into extension. The ability of a muscle to lengthen but to generate enough force to overcome resistance is what is qualified by the examiner and termed "Strong" or "Weak."

The grading system is based on muscle performance in relation to the magnitude of manual resistance applied by the examiner. Scores are ranked from no contraction to a contraction that can be performed against gravity and can accept "maximal" resistance by the examiner, depending on the size of the muscle and the examiner's strength. However, in the AK use of MMT the implication of grades is limited to an interpretation of 'better' or 'worse', 'stronger' or 'weaker,' and no assumption is made about the magnitude of difference between grades.

MMT procedures are also commonly employed in clinical neurology as a means of subjectively evaluating muscle function. The examiner in the application of force to the subject's resistance evaluates the muscle groups being studied as subjectively "weak" or "strong" on a 5-point scale [24].

MMT is employed by physical therapists to determine the grades of strength in patients with pathological problems and neurologic or physical injuries (strokes, post-polio syndromes, fractures, post-surgical disabilities, etc.). The physical therapist's patients are often initially examined by a medical doctor who supervises the physical therapist's rehabilitation programs that may involve isometric, isokinetic, and isotonic muscle training regimes for the gradual rehabilitation of muscle function (often involving instruments and machinery).

In the absence of a pathological neurological deficit (pathological deficits were originally what physicians sought to find using MMT), [25,26] clinical inferences are made based upon the result of the MMT. This method of MMT is used in both chiropractic and physical therapy to determine a patient's progress during therapy [3-9,20-23].

MMT, when employed by AK chiropractors, is used to determine whether manipulable impairments to neurological function (controlling muscle function) exist. For example, chiropractic management using MMT for a patient with carpal tunnel syndrome could involve assessment of the opponens policis and flexor digiti minimi muscles (innervated by the median and radial nerves), and then adjustment as indicated to the carpal bones, the radius and ulna, attention to an inhibited (on MMT) pronator teres muscle, adjustment of the cervical or thoracic spines, and evaluation of cranial nerve XI through MMT of the sternocleidomastoid and upper trapezius muscles. Any or all of these factors may require treatment in order to strengthen the inhibited opponens policis and flexor digiti minimi muscles that are evidence of the carpal tunnel syndrome. This "continuous nervous system" thinking and testing may allow the identification of contributing sites to a pain state.

The expectation in a chiropractic setting is that the proper therapy will immediately improve muscle strength upon MMT, taking the patient from "weak" to "strong." This is the reason that in most chiropractic settings, the grading system of muscle evaluation does not have as much significance as it does in physical therapy settings. Chiropractic therapy may produce rapid responses for the innervation of muscles because the basic therapy required for chiropractic patients is decompression of the nervous system. It is purported that this can be done readily with chiropractic manipulative therapy (CMT) [27-30].

When performed by an examiner's hands MMT may not be just testing for actual muscle strength; rather it may also test for the nervous system's ability to adapt the muscle to the changing pressure of the examiner's test. A nervous system functioning optimally will immediately attempt to adapt a muscle's activity to meet the demands of the test. There appears to be a delay in the recruitment of muscle motor units when the nervous system is functioning inadequately [66,71-73,82,90,102]. This delay varies with the severity of the nervous system's impairment, and influences the amount of weakness shown during the MMT.

Determining the ideal operational definition of a MMT can be difficult given the large number of test variations that exist. All of the tests described by Kendall, Wadsworth, Goodheart, Walther and others [3,20-23] involve multiple joint movements and handling techniques. This results in a large number of variables that are difficult to control.

Because of the variability possible during a MMT, several studies examining MMT have used specialized instrumentation to provide support for the extremity tested and for standardization of joint position. Throughout its history manual muscle testing has been performed by practitioners' hands, isokinetic machines, and other handheld devices. However, isokinetic machines and dynamometers for more objective testing of muscles are still too expensive or cumbersome for clinical use, but this equipment is useful for research purposes [20-23].

Kendall et al (1993) [21] state:

"As tools, our hands are the most sensitive, fine tuned instruments available. One hand of the examiner positions and stabilizes the part adjacent to the tested part. The other hand determines the pain-free range of motion and guides the tested part into precise test position, giving the appropriate amount of pressure to determine the strength. All the while this instrument we call the hand is hooked up to the most marvelous computer ever created. It is the examiner's very own personal computer and it can store valuable and useful information of the basis of which judgments about evaluation and treatment can be made. Such information contains objective data that is obtained without sacrificing the art and science of manual muscle testing to the demand for objectivity."

According to Walther (1988) [23]:

"Presently the best 'instrument' to perform manual muscle testing is a well-trained examiner, using his perception of time and force with knowledge of anatomy and physiology of muscle testing."

Regardless of the methods or equipment one uses to standardize MMT in a clinical or research setting, it is most important that the test protocol be highly reproducible by the original examiner and by others.

## Results

### Research on the Reliability of the MMT

One way researchers determine if a clinical test is consistent and repeatable over several trials is to analyze its reliability. The reliability of a diagnostic method is the consistency of that measurement when repeated. Depending on the type of measurement that is performed, different types of reliability coefficients can be calculated. In all coefficients, the closer the value is to 1, the higher the reliability. For instance, calculating Cohen's kappa coefficient allows the researcher to determine how much agreement existed between two or more doctors performing MMT on patients with low back pain. A value greater than .75 indicates "excellent" agreement, a value between .40 and .75 indicates "fair to good" agreement, and a value less than .40 indicates "poor" agreement [31]. The advantage of the kappa coefficient is that it is a measure of chance corrected concordance, meaning that it corrects the observed agreement for agreement that might occur by chance alone. There are difficulties with the interpretation of kappa and correlation coefficients that have been described by Feinstein and Brennan [32,33]. To examine the reliability coefficients calculated by the authors of MMT studies, see Table 1.

**Table 1:** Characteristics of 10 studies of the intraexaminer and interexaminer reliability of manual muscle testing (RCTs indicated by **)

**Authors, date**	**Subjects**	**Examiners**	**Design**	**Findings and statistics**
Pollard et al^55 ^(2005) **	106 volunteers	Novice examiner (5^th ^year) chiropractic student; experienced examiner (15 years MMT experience)	Interexaminer reliability of 2 common muscle tests	Deltoid muscle showed Cohen kappa value (k 0.62) and psoas muscle showed (k 0.67). Good interexaminer reliability shown between experienced and novice examiners.

Perry et al^43 ^(2004) **	16 patients with post-polio syndrome; 18 patients without pathology; 26 patients with signs of hip extensor weakness and post-polio syndrome	Several examiners	Supine MMT of hip extensor strength compared to strength values obtained by traditional prone test of hip extensor muscles in patients with post-polio syndrome	Reliability testing showed excellent agreement (82%). Subjects with pathology had significant differences in mean muscle torque (P < .01) strength. Predictive validity of MMT in patients with symptomatic post-polio syndrome affecting hip extensor muscles was excellent.

Escolar et al^56 ^(2001)	12 children with muscular dystrophy	12 novice and experienced examiners	To determine reliability of quantitative muscle testing (QMT, an instrument for measuring strength) compared to MMT	MMT was not as reliable among novice examiners as QMT. With adequate training of examiners an interclass correlation coefficient > 0.75 was achieved for MMT.

Caruso and Leisman ^36 ^(2000)	27 volunteers who knew nothing about MMT or AK	2 examiners	To show the difference between "weak" and "strong" muscles, using MMT and dynamometer testing	Study showed that examiners with over 5 years experience using AK had reliability and reproducibility (98.2%) when their outcomes were compared. Perception of "inhibition" or weakness made by examiner was corroborated by test pressure analysis using the dynamometer.

Florence et al ^47 ^(1992) **	102 boys aged 5 to 15 years.	Physical therapists	A double-blind, multicenter trial to document the effects of prednisone on muscle strength in patients with Duchenne's muscular dystrophy (DMD).	Reliability of muscle strength grades obtained for individual muscle groups and of individual muscle strength grades was analyzed using Cohen's weighted Kappa. The reliability of grades for individual muscle groups ranged from .65 to .93, with the proximal muscles having the higher reliability values. The reliability of individual muscle strength grades ranged from .80 to .99, with those in the gravity-eliminated range scoring the highest. Concluded that the MMT was reliable for assessing muscle strength in boys with DMD when consecutive evaluations are performed by the same physical therapist.

Barr et al ^42 ^(1991)	36 boys (11.7 +/- 3.9 years) with Duchenne or Becker muscular dystrophy.		Upper and lower extremities were evaluated by MMT for function, range of motion, and strength.	The data were analyzed using intraclass correlation coefficients (ICCs). For the interevaluator phase, ICCs for MMT was .90; For the intraevaluator phase, corresponding ICC was .80 to .96. Results confirm and extend observations by others that these assessment measures are sufficiently reliable for use in multiinstitutional collaborative efforts. These results can be used to design clinical trials that have sufficient statistical power to detect changes in the rate of disease progression.

Hsieh and Phillips ^46 ^(1990)	15 asymptomatic subjects	3 chiropractors	To determine the reliability of manual dynamometry using AK style of MMT, comparing doctor-initiated and patient-initiated MMT	Intratester reliability and correlation coefficients for testers 1, 2, and 3 were 0.55, 0.75, and 0.76 with doctor-initiated method; 0.96, 0.99, and 0.97 when patient-initiated MMT method. The intertester reliability coefficients were 0.77 and 0.59 on day 1 and 2 respectively for doctor-initiated method; and 0.95 and 0.96 for the patient-initiated method.

Wadsworth et al ^45 ^(1987)	5 muscle groups on 11 patients	physical therapists	To compare the intrarater reliability of MMT and hand-held dynamometer tests	The correlation coefficients were high and significantly different from zero for four muscle groups tested dynametrically and for two muscle groups tested manually. The test-retest reliability coefficients for two muscle groups tested manually could not be calculated because the values between subjects were identical. Concluded that both MMT and dynamometry are reliable testing methods, given the conditions described in this study.

Florence et al ^34 ^(1984)	Patients with Duchenne Muscular Dystrophy	physical therapists	To evaluate the (intraobserver) and (interobserver) reliability of MMT evaluation procedures to assess the efficacy of treatment of Duchenne muscular dystrophy.	Showed there was significant improvement in the degree of consistency of a given examiner's MMT scores when the examiner had more clinical experience and training in MMT. Author's concluded that MMT demonstrated reliability for an evaluation method that provided an objective foundation on which to claim if a drug or therapeutic procedure does or does not have an effect in treating Duchenne muscular dystrophy.

Jacobs ^44^(1981)	65 patients with suspected thyroid dysfunction	2 chiropractors	To compare AK diagnostic findings with laboratory findings	This double-blind study demonstrated an 81.9% agreement between two testers, indicating good inter-examiner reliability.

This review of the literature shows the importance of clinical experience and expertise, and this factor has been highlighted in many papers discussing the reliability of the MMT [20-23,34-36]. The skills of the examiners conducting studies on MMT and their skills in interpreting the derived information will affect the usefulness of MMT data. The examiner is obliged to follow a standardized protocol that specifies patient position, the precise alignment of the muscle being tested, the direction of the resisting force applied to the patient, and the verbal instruction or demonstration to the patient. All of these precautions have proven necessary to reliably study the validity of the MMT in the diagnosis of patients with symptomatology.

There was significant improvement in the degree of consistency of a given examiner's scores (as noted by Florence et al 1984) [34] when the examiner had more clinical experience and training in MMT. Mendell and Florence (1990) [35], Caruso and Leisman (2000), [36] and many other researchers of MMT have discussed the importance of considering the examiner's training on the outcomes of studies that assess strength via MMT [20-23].

Interexaminer reliability of the MMT has been reported by Lilienfeld et al (1954) [37], Blair (1955)[38], Iddings et al (1961) [39], Silver et al (1970) [40], Florence et al (1984) [34], Frese et al (1987) [41], Barr et al (1991) [42] and Perry et al (2004) [43]. Test-retest reliability has been examined by Iddings et al (1961), [39] Jacobs (1981) [44], Florence et al (1984) [34], Wadsworth et al (1987) [45], Mendell and Florence (1990) [35], Hsieh and Phillips (1990) [46], Barr et al (1991) [42], Florence et al (1992) [47], Lawson and Calderon (1997) [48], Caruso and Leisman (2000) [36], and Perry et al (2004) [43]. The levels of agreement attained, based upon +/- one grade were high, ranging from 82% to 97% agreement for interexaminer reliability and from 96% to 98% for test-retest reliability. The results of these studies indicate that in order to be confident that a true change in strength has occurred; MMT scores must change more than one full grade. In clinical research studies on chiropractic treatment, the change from an "inhibited" or "weak" muscle to a "facilitated" or "strong" muscle is a change in at least one full grade, and is a common result of successful treatment.

In the latter 11 studies, correlation coefficients are reported. These coefficients ranged from 0.63 to 0.98 for individual muscle groups, and from 0.57 to 1.0 for a total MMT score (comprised of the sum of individual muscle grades).

Using force measurements from both practitioner and patient, Leisman and Zenhausern demonstrated a significant difference in "strong" versus "weak" muscle testing outcomes and showed that these changes were not attributable to decreased or increased testing force from the practitioner performing the tests [49].

Table 1 provides a brief synopsis of several studies that investigated the reliability of MMT in both healthy and symptomatic subjects. The Table does not show the substantial amount of normative data that exists regarding muscle strength relating to patient age, position, tasks performed, and so on [51,52]. There also exists a large body of data demonstrating how electromyographic signals are used as an objective representation of neuromuscular activity in patients. The EMG is a valid index of motor unit recruitment and reflects the extent to which the muscle is active; however there are some difficulties with the sensitivity and specificity of electrodiagnosis [53]. All of these studies using MMT and instrumentation have collectively made a significant contribution to the study of neuromuscular function and represent different aspects of the muscular activity going on in patients.

### Research On the Validity of MMT

The next section of Results looks at the relationship between muscle strength as measured by MMT findings and the functional status of patients with a variety of symptoms.

Validity is defined as the degree to which a meaningful interpretation can be inferred from a measurement or test. Payton (1994) [58] states that validity refers to the appropriateness, truthfulness, authenticity, or effectiveness of an observation or measurement. In examining research studies and examination techniques using MMT and spinal manipulative therapy (SMT), clinicians need to become familiar with several different types of validity.

### Construct and content validity of MMT

Construct and content validity are two types of theoretical or conceptual validity. Generally, construct and content validity are proven through logical argument rather than experimental study. Construct validity is the theoretical foundation on which all other types of validity depend. Construct validity attempts to answer the questions, "Can I use this measurement to make a specific inference?" and "What does the result of this test mean?"

From the original work of Lovett (1915) [25,26] who developed MMT as a method to determine muscle weakness in polio patients with damage to anterior horn cells in the spinal cord, to the measurement of physical weakness from faulty and painful postural conditions, injuries, and congenital deformities [20-23,59,60], to neurologists who adopted MMT as part of their physical diagnostic skills, [24] to the use of MMT by some chiropractors beginning with AK technique to diagnose structural, chemical, and mental dysfunctions, the concept of manually examining the nervous system's status through MMT continues to evolve and gain adherents to this method [61]. The validity of Lovett's original MMT methods was based on the theoretical construct that properly innervated muscles could generate greater tension than the partially innervated muscles present in patients with anterior horn cell damage.

AK extends Lovett's construct and theorizes that physical, chemical, and mental/emotional disturbances are associated with secondary muscle dysfunction affecting the anterior horn of the spinal cord – specifically producing a muscle inhibition (often followed by overfacilitation of an opposing muscle and producing postural distortions in patients). Goodheart suggested, contrary to the physiotherapeutic understanding of the time, that muscle spasm was not the major initiator of structural imbalance [3,6]. According to Goodheart, the primary cause of structural imbalance is muscle weakness. Goodheart theorized that the primary weakness of the antagonist to the spastic muscle to be the problem. Muscle weakness (as observed by MMT) is understood as an inhibition of motor neurons located in the spinal cord's anterior horn motor neuron pool [62].

Chiropractic AK research has also suggested that there are five factors or systems to consider in the evaluation of muscle function: the nervous system, the lymphatic system, the blood vascular system, cerebrospinal fluid flow, and the acupuncture system [3,6].

Lamb states (1985) that MMT has content validity because the test construction is based on known physiologic, anatomic and kinesiologic principles [63]. A number of research papers have dealt with this specific aspect of MMT in the diagnosis of patients [64,65].

There have been a number of papers that have specifically described the validity of MMT in relationship to patients with low back pain. The correlation between "inhibited" or "weak" MMT findings and low back pain has been well established in the research literature. Several papers have shown that MMT is relevant and can be employed in a reliable way for patients with low back pain [63,66]. In a paper by Panjabi, it is proposed that the function of muscles, as both a cause and a consequence of mechanoreceptor dysfunction in chronic back pain patients, should be placed at the center of a sequence of events that ultimately results in back pain [67]. This paper argues that as a result of spinal dysfunctions (articular dysfunction, spinal lesions, and somatic dysfunction are terms also employed), muscle coordination and individual muscle force characteristics are disrupted, i.e. inhibited muscles on MMT. The injured mechanoreceptors generate corrupted transducer signals (that research suggests may be detected by EMG, dynamometers, and MMT), which lead to corrupted muscle response patterns produced by the neuromuscular control unit.

This article may be important for those in the manipulative professions who are evaluating the existence and consequences of spinal dysfunction. The key technical factor in this hypothesis would be the MMT that makes the detection of the muscular imbalances and spinal dysfunction cited by Panjabi identifiable. Another paper by Hodges et al (2003) suggests this hypothesis also [68]. Pickar has also shown there is a substantial experimental body of evidence indicating that spinal manipulation impacts primary afferent neurons from paraspinal tissues, immediately effecting the motor control system and pain processing [69].

Lund et al (1991) [70] reviewed articles describing motor function in five chronic musculoskeletal pain conditions (temporomandibular disorders, muscle tension headache, fibromyalgia, chronic lower back pain, and post-exercise muscle soreness). Their review concluded that the data did not support the commonly held view that some form of tonic muscular hyperactivity maintains the pain of these conditions. Instead, they maintain that in these conditions the activity of agonist muscles is often reduced by pain, even if this does not arise from the muscle itself. On the other hand, pain causes small increases in the level of activity of the antagonist. As a consequence of these changes, force production and the range and velocity of movement of the affected body part are thought to be reduced.

This paper describes with fascinating similarity one of the major hypotheses in MMT and chiropractic, namely that physical imbalances produce secondary muscle dysfunction, specifically a muscle inhibition (usually followed by overfacilitation of an opposing muscle). A paper by Falla et al (2004) described a similar model but involving patients with chronic neck pain [71]. A paper by Mellor et al (2005) presented this model in relationship to anterior knee pain [72], and Cowan et al (2004) in relationship to chronic groin pain with another paper demonstrating this mechanism in patellofemoral pain syndrome [73,74].

According to several studies, patients with low-back pain have lower mean trunk strength than asymptomatic subjects (Nummi et al 1978, Addison & Schultz 1980, Karvonen et al 1980, MacNeill et al 1980, Nordgren et al 1980, Mayer et al 1985, Triano 1987, Rantanen et al 1993, Hides et al 1996, Hodges et al 1996) [75-83]. Lifting strength is also decreased in persons disabled with chronic low-back pain (Chaffin & Park, 1973, Biering-Sorensen 1984, Mayer et al 1988) [84-86]. Pain itself is possibly a strength-reducing factor, as is the duration of back pain (Nachemson & Lindh 1969) [87].

These studies do not always clarify whether a muscle weakness or imbalance is primary or secondary to low-back pain. In spite of this, muscle weakness has frequently been cited as a primary factor in the etiology of low-back pain. (See Table 2) This is one of the bases on which Lamb argues that MMT has content validity [63].

**Table 2:** Characteristics of 8 Studies showing the prevalence of muscle dysfunction in patients with back pain (RCTs indicated by **)

**Authors, date**	**Subjects**	**Design**	**Findings and statistics**
Hossain et al ^90 ^(2005)	Literature review	Gait analysis studies reviewed show an orderly sequence of muscle activation – this contributes to efficient stabilization of the joint and effective weight transfer to the lower limb. Gluteus maximus fibres – lying almost perpendicular to the joint surfaces are oriented for this purpose. Biceps femoris is another important muscle that can also influence joint stability by its proximal attachment to sacrotuberous ligament.	Altered pattern of muscle recruitment has been observed in patients with low back pain. Because of its position as a key linkage in transmission of weight from the upper limbs to the lower, poor joint stability could have major consequences on weight bearing. It is proposed that sacro-iliac joint dysfunction can result from malrecruitment of gluteus maximus motor units during weight bearing, resulting in compensatory biceps femoris over activation. The resulting soft tissue strain and joint instability may manifest itself in low back pain. This thesis was also proposed by Janda (1964). ^18^

Falla et al ^71 ^(2004) **	10 patients with chronic neck pain; 10 controls	To compare activity of deep and superficial cervical flexor muscles during a test of craniocervical flexion.	Showed a strong linear relation between the electromyographic amplitude of the deep cervical flexor muscles and the incremental stages of the craniocervical flexion test for control and individuals with neck pain (P = 0.002). A reduced performance of the craniocervical flexion test is associated with dysfunction of the deep cervical flexor muscles.

Hodges et al ^83 ^(1996) **	15 patients with low back pain and 15 matched control subjects	Subjects performed rapid shoulder flexion, abduction, and extension in response to a visual stimulus. Electromyographic activity of the abdominal, and lumbar multifidus muscles recorded by surface electrodes.	Contraction of transversus abdominis was significantly delayed in patients with low back pain with all movements. The delayed onset of contraction of transversus abdominis indicated a deficit of motor control and is hypothesized to result in inefficient muscular stabilization of the spine.

Triano et al ^91 ^(1987) **	41 low-back pain patients; and 7 pain-free control subjects	To examine relations among some objective and subjective measures of low-back-related disability	Oswestry disability score related significantly (P less than 0.001) to presence or absence of relaxation in back muscles during flexion. Mean trunk strength ratios were inversely related to disability score (P less than .05). Findings imply that myoelectric signal levels, trunk strength ratios, and ranges of trunk motion may be used as objective indicators of low-back pain disability.

Biering-Sorensen ^85^(1984)	449 men and 479 women	The examination consisted of anthropometric measurements, flexibility/elasticity measurements of the back and hamstrings, as well as tests for trunk muscle strength and endurance.	The main findings were that good isometric endurance of the back muscles may prevent first-time occurrence of low back trouble (LBT) in men and that men with hypermobile backs are more liable to contract LBT. Weak trunk muscles and reduced flexibility/elasticity of the back and hamstrings were found as residual signs, in particular, among those with recurrence or persistence of LBT in the follow-up year.

McNeill T et al ^92 ^(1980) **	27 healthy males and 30 healthy females; and 25 male and 15 female patients with low-back pain and/or sciatica.	Maximum voluntary isometric strengths were measured during attempted flexion, extension, and lateral bending from an upright standing position.	The ratios showed that the patients with low back pain and/or sciatica had extension strengths that were significantly less than their strengths in the other types of movements tested. The strength ratios for attempted extension were particularly low for patients with sciatica. Both male and female with LBP and/or sciatica had approximately 60% of the absolute trunk strengths of the corresponding healthy subjects.

Karvonen et al ^77 ^(1980)	183 male conscripts. A history of sciatica was reported by 8%, lumbago by 13%, back injury by 13% and low back insufficiency by 63%.	To correlate muscle weaknesses in young men with complaints of LBP	Weak trunk extensors were associated with a history of sciatica; weak trunk flexors with back injuries and with current backache at work/exercise. Weak leg extensors showed associations with a history of low back insufficiency and of sick leave due to the back and with current hip pain. Men with a history of lumbago and of hip and knee complaints performed poorly during 12 min of running. The questionnaire and strength measurements proved suitable for studying low back syndrome in its early stages.

Addison et al ^76 ^(1980)	16 male and 17 female patients with chronic LBP	Maximum voluntary trunk strengths in the standing position were measured during attempted flexion, extension, and lateral bending. The trunk strengths of these patients were then compared with those of healthy subjects and with those of patients with low-back disorders who sought treatment as outpatients of a general orthopaedic office practice.	When compared with healthy subjects, the patients seeking hospitalization had significantly smaller strengths during attempted extension relative to their strengths during attempted flexion or lateral bending.

A number of general MMTs have been employed by all primary contact practitioners for the examination of patients with sciatic neuralgia. Dorsiflexion of the foot and the great toe, plantar flexion of the foot and great toe, quadriceps weakness, and peroneal muscle tests are each indicative of the status of the sciatic nerve and its branches [88,89].

To test the construct validity of these original hypotheses, researchers have attempted to quantify the muscle weakness that occurs with specific clinical conditions such as low back pain and soft tissue injuries. (See Table 2)

### The Convergent and Discriminant Validity of MMT

Convergent validity exists when a test, as predicted, demonstrates a strong correlation between two variables. Discriminant validity exists when the test, as predicted, demonstrates a low correlation between two variables. These tests, when found to have the proper correlations, lend support to the construct validity of the method of testing.

The convergent and discriminant validity of MMT was examined in a study by Jepsen et al (2006) [93]. They examined the relationship between MMT findings in patients with and without upper limb complaints. The examiners were blinded as to patient-related information, and examined 14 muscles in terms of normal or reduced strength. With a median odds ratio of 4.0 (95%CI, 2.5–7.7), reduced strength was significantly associated with the presence of symptoms.

Perry et al (2004) showed excellent convergent and discriminant validity of MMT in 16 patients with and 18 patients without post-polio syndrome pathology. Subjects with pathology showed significant differences in mean muscle strength (P < 0.01). The predictive validity of MMT in patients with symptomatic post-polio syndrome affecting the hip extensor muscles was found to be excellent [43].

Pollard et al (2006) also studied the convergent and discriminant validity of MMT in order to determine if a positive correlation of therapy localization to the "ileocecal valve point" producing weakness on MMT could predict low back pain in patients with and without low back pain [54]. The study also aimed to determine the sensitivity and specificity of the procedure. Of 67 subjects who reported low back pain, 58 (86.6%) reported a positive test of both low back pain and ICV point test. Of 33 subjects, 32 (97%) with no back pain positively reported no response to the ICV point test. Nine (9) subjects (13.4%) reported false negative ICV tests and low back pain, and 1 subject (3%) reported a false positive response for ICV test and no low back pain. Their results demonstrated that the low back pain group had significantly greater positive results (inhibited MMT) than those of the pain free group. Assuming this study is sound it may demonstrate the convergent validity of the method of MMT in relationship to patients with low back pain. The discriminant validity of MMT was shown in this study by its ability to find a low number of positive test results in the pain free groups. However, before accepting these results it would be important for them to be reproduced in another study.

Studies like the ones described above and later in this review (that examine whether MMT can discriminate between abnormal and normal spinal function and pain states) contribute to the evidence available to clinicians supporting the validity of MMT.

### Concurrent Validity of MMT

The concurrent validity of MMT has also been examined in several studies comparing strength scores obtained by MMT with strength readings obtained using quantitative instruments. The concurrent validity of a test refers to a test's ability to produce similar results when compared to a similar test that has established validity. The concurrent validity of the MMT would be examined when the MMT is compared to a "gold standard" confirmation diagnosis using EMG and/or dynamometer testing, for instance. Many studies have compared the findings of MMT with dynamometer tests favorably. (See Table 3)

**Table 3:** Characteristics of 8 studies examining the concurrent validity of MMT

**Authors, date**	**Subjects**	**Examiners**	**Design**	**Findings and statistics**
Bohannon ^95 ^(2001)	128 acute knee rehabilitation patients	physical therapist	To compare MMT with hand-held dynamometer measurements of knee extension strength	MMT and dynamometer scores highly correlated (r = 0.768; P < 0.001). Convergent and construct validity of MMT and dynamometry measurements demonstrated.

Caruso and Leisman^36 ^(2000)	27 volunteers with no knowledge about MMT or AK	2 examiners	To show the difference between "weak" and "strong" muscles, using MMT and dynamometer testing	Study showed that examiners with over 5 years experience using AK had reliability and reproducibility when their outcomes were compared. Perception of "inhibition" or weakness made by examiner was corroborated by test pressure analysis using the dynamometer.

Lawson and Calderon ^48 ^(1997) **	30 asymptomatic volunteers	Medical doctor	10 upper extremity muscles were tested using AK methods in double-blind conditions.	MMTs of "weak" or "strong" muscles showed significantly different electromyographic measurements and demonstrated a high correlation between testing methods.

Schwartz et al ^96 ^(1992)	122 patients with spinal cord injuries at C4–C6	physical therapists	Relationship between MMT and hand-head myometry compared	Sequential examinations with MMT and myometry were made at 72 hours, 1 week, and 2 weeks post-spinal cord injury and at 1, 2, 3, 4, 6, 12, 18, and 24 months post-injury. Results showed that 22 of 24 correlations between MMT and myometry were significant at p values less than .001.

Perot et al ^57 ^(1991)	10 subjects	Chiropractors	To measure and compare both electromyographic and MMT results after proprioceptive techniques to both strengthen and weaken muscles	Response of tibialis anterior muscle to proprioceptive technique showed a significant EMG difference that corresponded to the difference found between "strong" and "weak" MMT outcomes. AK proprioceptive procedure to reduce muscle tone found to correlate with MMT outcomes.

Hsieh and Phillips ^46 ^(1990)	15 asymptomatic subjects	3 chiropractors	To determine the concurrent validity of manual dynamometry using AK style of MMT, comparing doctor-initiated and patient-initiated MMT	Intratester reliability and correlation coefficients for testers 1, 2, and 3 were 0.55, 0.75, and 0.76 with doctor-initiated method; 0.96, 0.99, and 0.97 when patient-initiated MMT method. The intertester reliability coefficients were 0.77 and 0.59 on day 1 and 2 respectively for doctor-initiated method; and 0.95 and 0.96 for the patient-initiated method.

Wadsworth et al ^45 ^(1987)	5 muscle groups on 11 patients	physical therapist	To compare the concurrent reliability of MMT and hand-held dynamometer tests	The correlation coefficients were high and significantly different from zero for four muscle groups tested dynametrically and for two muscle groups tested manually. The test-retest reliability coefficients for two muscle groups tested manually could not be calculated because the values between subjects were identical. Conclusion that both MMT and dynamometry are reliable testing methods, given the conditions described in this study.

Bohannon ^97 ^(1986)	50 patients	physical therapist	To determine the relationship between MMT word scores and dynamometer force scores using Kendall tau.	MMT scores and dynamometer test scores were significantly correlated (p less than 0.001). Percentage MMT and dynamometer test scores were significantly different (p less than 0.001). These results suggest that the two procedures measure the same variable-strength.

Marino et al ^50 ^(1982)	128 patients	physical therapists	To compare MMT findings with hand-held dynamometer (HHD) findings, with precise repetition of the MMT	The MMT and HHD values were within 5% of each other. The average hip abduction and hip flexion scores measured by the HHD were consistent with the examiner's perception of muscle weakness (P less than 0.001).

Triano and Davis ^98 ^(1976)	10 patients with "reactive muscle" phenomena described in AK	chiropractor	In patients with reactive muscle pairs (between the rhomboid and deltoid muscles), EMG and MMT findings were compared.	Study demonstrated that the reactive muscle phenomenon is, in fact, a physiologic imbalance of muscle measurable by EMG and MMT and was not a psychologic suggestion or an overpowering of the tested arm by brute force. These data showed that the deltoid-rhomboid "reactive muscle" represents a real physiological phenomenon.

Marino et al (1982) [50] and Wadsworth et al (1987) [45] showed significant reliability between handheld dynamometers and MMT. Scores measured with the dynamometers were consistent with the examiner's perception of muscle weakness (P less than 0.001) in both studies.

Leisman et al (1995) showed that chiropractic muscle testing procedures could be objectively evaluated through quantification of the electrical characteristics of muscles, and that the course of chiropractic treatments can be objectively plotted over time [49].

The use of EMG or dynamometers as a gold standard is arguable however because false positive or negative findings may exist, and these instruments measure different aspects of muscular activity [20]. Even the MRI (another diagnostic "gold standard") has been found to lack sensitivity and specificity. MRI can identify a lesion but cannot detail the relationship of the finding with the patient's symptoms [94].

There is increasing demand for objectivity in regard to muscle testing measurements. Electromyograms are expensive machines, and setting patients up on the machines in the clinical setting is time-consuming. A review of the literature on dynamometers reveals some of the problems associated with their use. These include problems with the actual forces measured by a hand-held dynamometer (HHD); providing the stabilization that is essential for controlling variables and for standardization of the testing technique; as even a slight tipping of the devise during testing can alter its results [20-22,93]. These are important factors when considering the cost-effectiveness and clinical usefulness of these other testing procedures for muscle strength assessment.

### Predictive Validity and Accuracy of the MMT

A second form of validity is called predictive validity. Comparing a test to supporting evidence that is obtained at a later date assesses predictive validity.

The accuracy of a diagnostic test is usually determined by examining the ability of the test to assist clinicians in making a correct diagnosis. A good diagnostic test minimizes the probability of the clinician finding a positive response in healthy people and negative test results in people with dysfunction or pathology. A good diagnostic test therefore minimizes the probability of either a false positive or a false negative result. The accuracy of the test is defined as the probability that people who truly should have the positive response receive a positive response when the test is performed. The accuracy of the test is also defined as the probability that people who should truly have a negative response correctly receive a negative response when the test is performed.

Table 4 provides a brief summary of several studies that examine the presence of positive MMT in suspected disorders of neural origin.

**Table 4:** Characteristics of 14 studies examining the Clinical Relevance, Predictive Validity and Accuracy of MMT (RCTs indicated by **)

**Authors, date**	**Diagnosis**	**Subjects**	**Repeated Observations**	**Treatments**	**Outcomes**
Jepsen et al ^93 ^(2006) **	Upper limb pain	41 patients, 19 with upper limb pain	MMT, with examiners blinded as to patient pain status.	None	Reduced strength of upper limb muscles was significantly associated with the presence of symptoms. A median odds ratio of 4.0 (2.5–7.7).

Pollard et al ^54 ^(2006) **	Low back pain	67 of 100 patients have low back pain	MMT with therapy localization to an Ileo-cecal valve reflex point	None	Of 67 subjects who reported low back pain, 58 (86.6%) reported a positive test of both low back pain and ICV point test. Of 33 subjects, 32 (97%) with no back pain positively reported no response to ICV point test. Nine (9) subjects (13.4%) reported false negative ICV tests and low back pain, and 1 subject (3%) reported a false positive response for ICV test and no low back pain. The ileocecal valve test as a diagnostic measure of low back pain was found to have excellent measures of sensitivity, specificity and diagnostic competency.

Niemuth et al ^99 ^(2005) **	Single leg overuse injury	30 recreational injured runners (17 female, 13 male) and 30 noninjured runners (16 female, 14 male) served as controls.	Muscle strength of the 6 major muscle groups of the hip was recorded using a hand-held dynamometer.	None	No significant side-to-side differences in hip group muscle strength were found in the noninjured runners (P = 0.62–0.93). Among the injured runners, the injured side hip abductor (P = 0.0003) and flexor muscle groups (P = 0.026) were significantly weaker than the noninjured side. In addition, the injured side hip adductor muscle group was significantly stronger (P = 0.010) than the noninjured side.

Michener et al ^64 ^(2005)	Shoulder pain	40 patients with shoulder pain and functional loss	Hand held dynamometer testing performed as MMT for the lower trapezius, upper trapezius, middle trapezius, and serratus anterior muscles. Concurrently, surface electromyography (sEMG) data were collected for the 4 muscles. The same procedures were performed 24 to 72 hours after the initial testing by the same tester.	None	Intraclass correlation coefficients for intratester reliability of measurements of isometric force obtained using an HHD ranged from .89 to .96. The standard error of the measure (90% confidence interval [CI]) ranged from 1.3 to 2.7 kg; the minimal detectable change (90% CI) ranged from 1.8 to 3.6 kg. Construct validity assessment, done by comparing the amounts of isometric muscle activity (sEMG) for each muscle across the 4 muscle tests, revealed that the muscle activity of the upper trapezius and lower trapezius muscles was highest during their respective tests.

Moncayo et al ^100 ^(2004)	Thyroid associated orbitopathy (TAO)	32 patients with TAO, 23 with a long-standing disease, and 9 showing discrete initial changes	Positive TL (patient touches area of dysfunction and weakening occurs on MMT) reactions were found in the submandibular tonsillar structures, the tonsilla pharyngea, the San Yin Jiao point, the lacrimal gland, and with the functional ocular lock test of AK.	AK treatment and homeo-pathic remedies	Change of lid swelling, of ocular movement discomfort, ocular lock, tonsil reactivity and Traditional Chinese Medicine criteria including tenderness of San Yin Jiao (SP6) and tongue diagnosis were improved. Clinical trial of 3–6 months showed all relevant parameters improved.

Rainville et al ^101 ^(2003)	sciatica	33 patients with L3 or L4 radiculopathy; 10 patients with L5 or S1 radiculopathy	To test quadriceps strength with MMT	None	Knee flexed MMT weakness of the quadriceps showed kappa coefficient of (0.66). Patients with radicular pain caused by L5 or S1 could perform the quadriceps test. Weakness of quadriceps correlated with L3 or L4 radiculopathy.

Great Lakes ALS Study Group ^65 ^(2003)	amyotrophic lateral sclerosis (ALS)	63 patients with ALS	Compared test reliability of MMT and maximal voluntary isometric contraction (MVIC) scores among institutions and test validity by comparing change over time between MMT and MVIC.	None	Reproducibility between MVIC and MMT was equivalent. Sensitivity to detect progressive weakness and power to detect this change, however, favored MMT. In multicentered trials, uniformly trained physical therapists reproducibly and accurately measure strength by both MMT and MVIC. The authors found MMT to be the preferred measure of global strength because of its better Pearson correlation coefficients, essentially equivalent reproducibility, and more favorable coefficient of variation.

Nadler et al ^66 ^(2001)	13 college athletes with low back pain	Of 163 athletes (100 male, 63 female), 5 of 63 females and 8 of 100 males required treatment for LBP.	A dynamometer incorporated into a specially designed anchoring station was used for testing the hip extensors and abductors. The maximum force generated for the hip abductors and extensors was used to calculate a percentage difference between the right and left hip extensors and abductors.	athletic trainers for LBP unrelated to blunt trauma over the ensuing year	Logistic regression analysis indicated that for female athletes, the percentage difference between the right and left hip extensors was predictive of whether treatment for LBP was required over the ensuing year (P = 0.05). Validity shown that hip muscle imbalance is associated with LBP occurrence in female athletes. Research supports the need for the assessment and treatment of hip muscle imbalance in individuals with LBP.

Monti et al ^103 ^(1999)	None	89 healthy college students	To determine the differences in MMT outcomes after exposure to congruent and incongruent semantic stimuli	None	Approximately 17% more total force over a 59% longer period of time could be endured when subjects repeated semantically congruent statements (p < .001). Over all, significant differences were found in muscle test responses between congruent and incongruent semantic stimuli.

Schmitt et al ^104 ^(1998)	Allergies	17 subjects	To determine whether subjective muscle testing employed by Applied Kinesiology practitioners, prospectively determine those individuals with specific hyperallergenic responses.	None	Each subject showed muscle-weakening (inhibition) reactions to oral provocative testing of one or two foods for a total of 21 positive food reactions. Tests for a hypersensitivity reaction of the serum were performed using both a radio-allergosorbent test (RAST) and immune complex test for IgE and IgG against all 21 of the foods that tested positive with A.K. muscle screening procedures. These serum tests confirmed 19 of the 21 food allergies (90.5%) suspected based on the applied kinesiology screening procedures.

Goodheart ^105 ^(1990)	Imbalanced weight bearing on right and left feet	40 patients	40 patients were evaluated for pre- and post-treatment weight balance.	AK examin-ation and treatment	Of the 40 patients, only one had minimal changes in weight upon two scales beneath the feet when both flexing and extending the spine.

Jacobs et al ^44 ^(1984)	Thyroid dysfunction	65 patients	Patients evaluated for thyroid dysfunction by MMT, and laboratory testing.	AK and labor-atory examina-tion	MMT ratings correlated with clinical ratings (r_s _= .36, p < 0.002) and with laboratory ratings (r_s _= .32, p < 0.005). Correlation between clinical and laboratory diagnosis was .47, p < 0.000. Three AK TL points had a significant correlation with the laboratory diagnosis (p < .05). "AK enhanced but did not replace clinical/laboratory diagnosis of thyroid dysfunction."

Scoop ^106 ^(1979)	Allergy	10 subjects	Subjects with unilateral weak muscles were given either a placebo or the nutrient that is hypothesized in AK to be associated with the muscle. Muscle tone was measured by a Jaymar dynamometer and with AK MMT methods.	Nutrition	The increase in muscle tone approximately 10 seconds after ingestion was 21% for the nutrient group and was a statistically significant (p < 0.05) increase in comparison with the placebo group. In the cerebral allergy testing part of the study, a 15% decrease in muscle tone of the pectoralis major clavicular was used as the criterion for cerebral allergy. The muscle testing method was then compared to results obtained by a Philpott-type fast with progressive reintroduction of foods. Correlation between foods identified as provocative by muscle testing and by the fast was .81. Observation of clinical results obtained with muscle testing suggests the method has substantial clinical utility. Pearson Product-Moment Correlation between testers was .91, suggesting that muscle testing is reliable between testers.

Carpenter et al ^107 ^(1977)	None	80 students	The muscles hypothesized in AK to be associated with certain organs were tested with an instrument after irritation of the related organs. Then a control muscle was tested. 4 organ muscle associations were evaluated: the eye, ear, stomach, and lung. The stomach was irritated by placing cold water into it; the eye with chlorinated water; the ear with sound of a controlled frequency and decibel rate; and the lung with cigarette smoke.	None	In 80 subjects, a total of 139 organs were irritated. In all cases, the associated muscle weakened significantly after the irritation. The control muscle also weakened, but to a much lesser degree.

### The Emerging Construct in the Research on MMT

In order to evaluate the scientific merit of MMT we have discussed the importance of the operational definitions, reliability and validity in MMT research. The original construct of the MMT was that it documented impairments in muscle strength. Muscle inhibitions (detected by MMT) are understood in chiropractic and AK to be reflective of an inhibition of motor neurons located in the spinal cord's anterior horn motor neuron pool as a result of dysfunction involving one or more of the "5-factors of the IVF" [3-9,62].

A complication to the original construct of MMT from Lovett and others has emerged with the increasing awareness that the responses to the MMT are not solely due to the denervation effects on neural tissues in conditions like polio, but also co-existing inputs to the spinal cord's anterior horn and the processing state of the CNS [62]. Chiropractic research and anecdotal evidence from clinical practice have also suggested that there are five factors or systems to consider in the evaluation of muscle function: the nervous system, the lymphatic system, the vascular system, cerebrospinal fluid flow, and the acupuncture system [3-9,62]. Chiropractic clinical experience and research has also suggested that dysfunction in a muscle may be caused by a failure of any of these systems and that the MMT response may provide important clues regarding the origin of that dysfunction. Applying the proper manipulative therapy may then result in improvement in the inhibited muscle, pain, movement and posture. (See Table 5) However RCTs and other substantive research studies are required before we can assert with confidence the relevance of each of these factors.

**Table 5:** Characteristics of 19 case reports of positive experiences for patients (n = 1 – 88) treated with chiropractic AK technique

**Authors, date**	**Diagnosis**	**Subjects**	**Repeated Observations**	**Treatments**	**Outcomes**
Cuthbert ^109 ^(2006)	Motion sickness disorder	1: 66 yoa female 2: 45 yoa female 3: 9 yoa female	Proprioceptive testing (Freeman-Wyke and Hautant's tests), AK MMT and palpation	Spinal and cranial chiropractic manipulative therapy (CMT)	1: Able to drive car and ride in a boat and airplane symptom free after 4 visits. 2: Able to drive car symptom free after 6 visits. 3: Able to drive in car symptom free after 4 visits

Cuthbert et al ^110 ^(2005)	optic nerve neuritis exacerbated by an Arnold-Chiari malformation (Type I) of the cerebellum	1: 20 yoa female	AK MMT to diagnose vertebral subluxations and cranial lesions; ocular muscle testing, TMJ testing	Cranial and spinal CMT	Patient had lost her vision in the right eye 3 weeks previous to treatment. After 1 visit, patient could see 20–30 on Snellen eye chart. Visual acuity 20-13 after 3^rd ^visit and asymptomatic 3 years later.

Meldener ^111 ^(2005)	Post-surgical hip dislocation	1: 75 yoa male	AK MMT to diagnose muscular weakness around hip and throughout the body	AK and CMT therapy, focusing on the connection of the TMJ and occlusion to instability of the hip	No hip dislocation since vertical dimension was increased with new upper dentures on doctor's recommendation.

Chung et al ^112 ^(2005)	Dental occlusion problems	7: male 3: female	AK MMT during application of an oral dental appliance	None	AK MMT reliable and repeatable on different days. MMT useful to locate the kinesiologic occlusal position for the fabrication of an oral appliance to treat TMJ disorders.

Caso ^113 ^(2004)	congenital bowel abnormality related to low back pain.	1: 29 yoa male	AK MMT to diagnose large bowel dysfunction	CMT and stimulation of Chapman's reflex points by the doctor and the patient at home	Resolution of the patient's low back pain as well as improved bowel function.

Moncayo et al ^100 ^(2004)	Thyroid associated orbitopathy (TAO)	32 patients with TAO, 23 with a long-standing disease, and 9 showing discrete initial changes	Positive TL (patient touches area of dysfunction and weakening occurs on MMT) reactions were found in the submandibular tonsillar structures, the tonsilla pharyngea, the San Yin Jiao point, the lacrimal gland, and with the ocular lock test of AK.	AK treatment and homeopathic remedies	Change of lid swelling, of ocular movement discomfort, ocular lock, tonsil reactivity and Traditional Chinese Medicine criteria including tenderness of San Yin Jiao (SP6) and tongue diagnosis were improved. Clinical trial of 3–6 months showed all relevant parameters improved.

Cuthbert ^114^(2003)	Down syndrome	15 children	Informal report by the parents of child's function and health status.	CMT to the spine and cranium, with nutritional support as needed.	Improved fine motor skills; use of the hands and fingers; ability to crawl bilaterally with arms and legs; ability to stand and walk; decrease in tongue thrusting; problems with ears and sinuses were all improved in function as noted by parents, teachers, and doctor.

Maykel ^115 ^(2003)	Blocked naso-lacrimal canal	1: 14-month male	AK MMT and informal report of child's function and health status by the parents.	CMT to the spine and cranium	Child treated 5 times over a 6-week period with resolution of his eye problem.

Weiss ^116 ^(2003)	Menstrual difficulty and exhaustion	1: 39 yoa female	AK MMT and patient report of condition	Nutritional counseling and CMT to the spine and cranium	Treatment to the sacrococcygeal area with cranial correction and nutritional support improved her energy level and cycling performance.

Sprieser ^117 ^(2002)	Episodic paroxysmal vertigo	1: 17 yoa female	AK MMT and patient report of her condition	CMT to the spine and cranium as well as AK/meridian therapy techniques	After 4 treatments and 3 other treatments by a Qi-Gong master the patient remained free of any vertigo at 3 year follow up.

Leaf ^118 ^(2002)	Severe equilibrium problems	1: 48 yoa female	AK MMT and patient report of condition	Cervical traction of 6 pounds while patient walked for 15 minutes	After cervical traction-distraction patient was able to stand with her feet together with no body sway and displayed no signs of nystagmus.

Gregory et al ^119 ^(2001)	women with moderate to severe breast pain	88: females, predominantly premenopausal, with cyclical and non-cyclical breast pain	AK MMT to diagnose neurolymphatic reflex dysfunction of the large intestine	CMT and stimulation of Chapman's reflex points by the doctor and the patient at home	Immediately after treatment there was considerable reduction in breast pain in 60% of patients with complete resolution in 18%. 2 months after initial treatment, there was a reduction in severity, duration and frequency of pain of 50% or more in 60% of cases (P < 0.01).

Cuthbert ^120 ^(2001)	Bell's Palsy	1: female	AK MMT to diagnose cranial, cervical, TMJ, and muscular imbalances	CMT to the spine, TMJ, and cranium	Complete resolution of facial nerve palsy after 6 visits over 14 days.

Calhoon ^121 ^(2001)	Multiple sclerosis	1: 43 yoa female	AK MMT and patient report of condition	CMT to the spine, TMJ, and cranium and nutritional support	26 months after initial visit patient had regained her ability to write and could shower without assistance for the first time in 2 years.

Mathews et al ^122 ^(1999) **	Learning disabilities	10 children compared with a control group of 10 children matched for age, IQ and social background that had not received any treatment over a similar period.	AK MMT examination and sensory challenges; the children were tested before and after treatment by an Educational Psychologist using standardized tests of intelligence to monitor changes in their learning skills.	AK treatment	Educational psychologist's testing demonstrated children treated with AK had an improvement in their learning abilities during the course of 9 to 12 treatment sessions during a period of 6–12 months.

Masarsky et al ^123 ^(1991)	Somatic dyspnea	6: males and females	AK MMT examination methods; forced vital capacity (FVC) and forced expiratory volume in one second (FEV-1) measurements pre- and post-treatment (post-treatment measurements taken 3 days later to 1 month later).	AK treatment including neurolymphatic and neurovascular reflexes were employed for the diaphragm muscle; evaluation of the meridian system; cranial manipulation (AK methods); and treatment for inhibited muscles involved in respiration.	All patients reported improvement in their breathing difficulty. 4 of the 6 patients also had improved FCV and FEV-1 between 0.1 and 0.8 liters.

Goodheart ^105 ^(1990)	Imbalanced weight bearing on right and left feet	40 patients	40 patients were evaluated for pre- and post-treatment weight balance.	AK examination and treatment	Of the 40 patients, only one had minimal changes in weight upon two scales beneath the feet when both flexing and extending the spine.

Masarsky et al ^124^	Chronic obstructive pulmonary disease	1: male	AK MMT examination methods; forced vital capacity (FVC) and forced expiratory volume in one second (FEV-1) measurements pre- and post-treatment, covering an 8-month period.	AK examination and treatment	Improvements were noted in forced vital capacity, forced expiratory volume in one second, coughing, fatigue, and ease of breathing (sign significant at 0.005 level). Improvement was also noted in laryngospasm.

Jacobs et al ^44 ^(1984)	Thyroid dysfunction	65: males and females	Patients evaluated for thyroid dysfunction by AK and laboratory testing	None	AK ratings correlated with laboratory ratings (r_s _= .32, p < .002) and with laboratory ratings (r_s _= .32, p < .005). Correlation between clinical and laboratory diagnosis was .47, p < .000. 3 AK therapy localizations had a significant correlation with the laboratory diagnosis (p < .05). AK enhanced but did not replace clinical/laboratory diagnosis of thyroid dysfunction. Evidence indicated a significant correlation between certain AK tests and an elevated LDH in the serum.

To be valid in this new model the MMT would have to reliably sample components of both the central and peripheral nervous systems and be performed in the context of a new, more holistic conceptual model of functional neurology. The future of chiropractic MMT research will depend upon demonstrating the validity and reliability of the MMT for evaluating these types of dysfunctions affecting the anterior horn motor neuron pool.

Understanding normal neuromuscular mechanisms is essential to identifying abnormal and also being able to physically test them. In this way the practitioner may be able to specifically determine areas of dysfunction and thereby individualize the treatment given. More importantly, MMT may allow the neuromuscular system to be used interactively (by examiner and patient) and as a key element in the assessment and treatment of the functional disorders of the patient. This ability to "manipulate" the neuromuscular system, with an aim of changing the patient's muscular function, postural balance and strength, and to measure the outcome is conceptually an important component of the chiropractic and AK approach to health care. If a patient's injury causes pain and dysfunction, an effective therapy may not only be in the elimination of pain but also an improvement in muscle function as evidenced by the same method of assessment originally used to diagnose the problem. This may add an important measure of objectivity to clinical practice, and potentially increase a patient's awareness about their body and their body's ability for improvement as a result of the therapy given.

To provide the strongest evidence for the use of chiropractic MMT techniques, more randomized controlled clinical trials (RCTs) and systematic reviews will be essential. Although RCTs will be required to document a cause-effect relationship between treatment and outcome, they are frequently impractical projects for the practicing clinician. This is frustrating because it is the clinician who depends on scientific proof that these techniques work.

One alternative is for groups such as ICAK and those who use AK and MMT methods to organize and fund these RCT's. Work so far in this area remains largely limited to reliability and observational studies. Unfortunately, there have not been significant efficacy studies in this area, nor have there been many significant efficacy studies conducted in the chiropractic research arena in general [108].

Nineteen examples of peer-reviewed published case reports using MMT and chiropractic AK protocols are presented in Table 5. These 19 case studies demonstrate how the practicing clinician may help narrow the gap between practice and research.

Although case reports cannot prove a treatment's effectiveness, they can describe the performance of techniques in a way that can initiate an hypothesis for a future RCT. More case reports may also add to the body of knowledge in the field of chiropractic AK and MMT.

## Conclusion

After 42 years of development and research, the chiropractic profession's use of MMT and AK chiropractic technique has become one of the many diagnostic methods from which some doctors of chiropractic draw their clinical procedures.

In the last forty years we have become more aware of the nervous system. This awareness has allowed us to evaluate patients more completely and from an integrated neuromuscular perspective. This holistic system of approach for the evaluation of neuromuscular function continues to be updated on a regular basis with new and exciting research. Much of the evaluation and treatment of patients using MMT and manual methods remains and will always remain an art. However, we must provide these artistic endeavors with a solid scientific foundation.

Although this narrative literature review offers considerable evidence about the reliability and validity of MMT as an examination tool, most of the rigorous, systematic research on this form of examination has emerged in just the past 30 years. Although evaluation of patients using MMT methods have been investigated with RCTs, prospective (cohort) studies, retrospective studies, single-subject case series and case reports, many questions about the MMT remain unanswered.

One shortcoming is the lack of RCTs to substantiate (or refute) the clinical utility (efficacy, effectiveness) of chiropractic interventions based on MMT findings. Also, because the etiology of a muscle weakness may be multifactorial, any RCT that employs only one mode of therapy to only one area of the body may produce outcomes that are poor due to these limitations.

A limitation of this review may involve research published outside the main databases searched, as well as research articles involving some form of muscle testing but not using the terms *manual muscle test*, *manual muscle testing*, or *applied kinesiology *as they may not have been accessed and included here. In addition this paper has not critically rated each study for its internal and external validity. Such a systematic review should be the subject of future research.

Throughout this paper we have tried to answer the question, "Are AK and MMT worthy of scientific merit?" In order to evaluate the effectiveness of MMT in the diagnosis of patients with musculoskeletal and nervous system problems, it is necessary to survey the full range of research studies that have addressed the topic, giving due consideration to the strengths and weaknesses of the studies in the literature.

Hopefully this literature review has stimulated a desire for others to review the current MMT literature and become an effective user of and contributor to chiropractic MMT research [125,126].

## Competing interests

SCC is a Board Member for the International College of Applied Kinesiology (I.C.A.K.). GJG is the Research Director and Founder of the I.C.A.K. SCC and GJG both employ MMT and AK methods in their evaluation and treatment of patients.

## Authors' contributions

SCC and GJG conceived the research idea. SCC acquired the papers and constructed the literature review. SCC and GJG critically appraised the studies. SCC and GJG drafted the manuscript and approved the final version for publication.
